#  The Reproducibility and Comparative Validity of a Non-Nutritive Sweetener Food Frequency Questionnaire

**DOI:** 10.3390/nu10030334

**Published:** 2018-03-10

**Authors:** Emily A. Myers, Erin M. Passaro, Valisa E. Hedrick

**Affiliations:** Department of Human Nutrition, Foods, and Exercise, Virginia Polytechnic Institute and State University, 295 West Campus Drive, Blacksburg, VA 24061, USA; eamyers@vt.edu (E.A.M.); eperin92@vt.edu (E.M.P.)

**Keywords:** dietary assessment, non-nutritive sweeteners, food-frequency questionnaires, reproducibility, validity

## Abstract

In order to better assess non-nutritive sweetener (NNS) consumption, measurement tools with greater utility are needed. The objective of this investigation is to determine the reproducibility and validity of a newly developed NNS food frequency questionnaire (NNS-FFQ) that measures five types of NNS (saccharin, aspartame, acesulfame potassium, sucralose and erythritol). Adult participants (*n* = 123, 56% female, 75% Caucasian, mean age = 36.8 ± 16.6) completed the NNS-FFQ twice and had 24-h dietary recalls three times over a two-week study period. Reproducibility between two administrations of the NNS-FFQ was assessed via Bland–Altman plots, Spearman’s correlations (*r_s_*) and paired samples *t*-tests. Bland–Altman plots, Cohen’s κ, Spearman’s correlations (*r_s_*), and paired samples *t*-tests compared NNS intake between the two methods for validity. For reproducibility analyses, Bland–Altman analyses revealed agreement levels above the 95% acceptance level for total NNS (99.2%), erythritol (99.2%), and aspartame (96.7%). Agreement levels for acesulfame potassium (94.3%), saccharin (94.3%), and sucralose (94.3%) were slightly below the acceptable level. For validity analyses, Bland–Altman analyses revealed agreement levels above the 95% acceptance level for total NNS (95.1%), sucralose (95.9%), saccharin (95.9%), and erythritol (95.1%). Agreement levels for aspartame (94.3%) and acesulfame potassium (92.7%) were slightly below the acceptable level. Although less than desirable agreement was found between the methods for aspartame and acesulfame potassium, some variance was expected due to the habitual nature of the NNS-FFQ as compared to the recent intake reported by recalls. Within the context of this constraint, the NNS-FFQ demonstrates acceptable reproducibility and validity. The NNS-FFQ is a brief questionnaire that could be administered among diverse participants at the individual and population levels to measure habitual NNS intake.

## 1. Introduction

Non-nutritive sweeteners (NNS), or artificial sweeteners, are substances that have a concentrated sweet taste within a very small amount of the substance. NNS are frequently debated for their role among functional foods [1]. These low-calorie intense sweeteners have been promoted for their potential to reduce added sugar consumption, facilitate weight loss, and control blood glucose levels [2,3,4]. However, they have also been investigated for their potential negative side effects, such as cancer, insulin resistance, and compensatory appetite [5,6,7,8,9]. 

These “high-intensity sweeteners” are between 160 and 1000 times sweeter than sucrose [3]. While some of these sweeteners do contain calories, the amount is negligible due to the very small amount needed to provide a sweet flavor. In the United States (US), the Food and Drug Administration (FDA) regulates NNS as food additives with six NNS that are currently FDA approved: saccharin, aspartame, acesulfame potassium, neotame, advantame, and sucralose [10]. When a food additive has been “adequately shown to be safe” by qualified experts, it is given the label “Generally Recognized As Safe” or GRAS [11]. GRAS labels can be determined by companies without informing the FDA and GRAS products do not require premarket approval from the FDA [10]. Stevia and luo han guo, also known as monk fruit, are currently classified as GRAS by the FDA, but have not been evaluated for FDA approval as food additives [10]. In Europe, NNS are evaluated and regulated by the European Food Safety Authority’s Panel on Food Additives and Nutrient Sources Added to Food (ANS Panel). There are eight NNS approved for use in Europe, acesulfame potassium, aspartame, cyclamate, neohesperidin dihydrochalcone, saccharin, sucralose, thaumatin, and stevia [12,13]. 

There are many potential associations between NNS consumption and health outcomes that are yet to be investigated; however, the lack of an accurate and rapid method for measuring NNS intake hinders the advancement of this topic. The majority of investigations exploring the impact of NNS intake look at NNS intake as a whole or use diet soda as a proxy for NNS consumption [14]. This approach has inherent issues, as it is not a valid assumption that all NNS have equal impacts on health outcomes. Without the ability to accurately and quickly measure habitual NNS consumption (total and specific types), it is difficult to assess the true impact of NNS on the health of consumers. The availability of such a method would advance knowledge related to how much NNS individuals consume, as well as amounts of specific types of NNS; and thus, allow researchers to inferentially examine potential associated health outcomes. 

Food frequency questionnaires (FFQ) may potentially fill this gap. FFQ are subjective dietary assessment tools that measure habitual consumption within certain food or beverage categories over various time periods [15,16]. FFQ can be fully-quantitative, measuring how often and how much of each item on a list is consumed [17], or semi-quantitative, measuring only how often each item on a list is consumed [15]. The National Institute of Cancer Division of Cancer Control and Population Sciences currently reports 146 validated FFQ [18]. To address limitations related to NNS research, valid and reproducible measurement tools need to be developed to better assess NNS consumption, including amounts consumed of specific NNS types. Thus, the objective of this investigation is to determine the reproducibility and comparative validity of a newly developed fully-quantitative NNS-FFQ that can quickly assess NNS consumption in approximately 5–10 min, as compared to multiple 24-h dietary recalls, which could take 20–30 min to collect the information from the participant, plus nutrient analysis time. 

## 2. Materials and Methods

### 2.1. Subjects and Design

Adult participants residing in southwest Virginia (*n* = 125) were recruited to participate in this observational study, with 123 participants being included in the final analysis after possible outliers were removed. Eligible participants were English-speaking adults aged 18 years or older. Participants were recruited through traditional methods, including flyers and listservs. This study was conducted according to the guidelines laid down in the Declaration of Helsinki and all study procedures involving human subjects were approved by the Virginia Tech Institutional Review Board (IRB #15-682, approved 25 September 2015). Participants provided written informed consent before enrollment.

**Figure 1:**
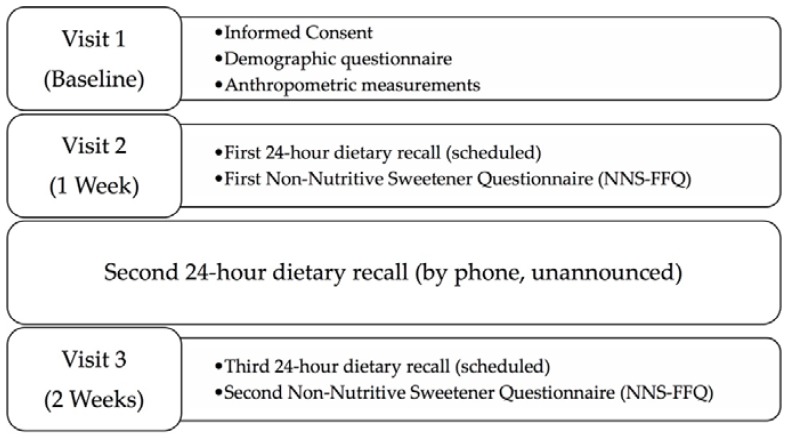
Study design and timeline.

Participants completed three visits over the course of two weeks (Figure 1). Measures included the collection of three 24-h dietary recalls and the completion of the NNS-FFQ twice. During the first visit to the laboratory, participants provided demographic information, height, without shoes, was measured in centimeters using a research-grade digital stadiometer, and weight, in light clothing without shoes, was measured to the nearest 0.1 kg using a calibrated digital Tanita scale (Model: TBF-310GS; Tokyo, Japan). During the second visit, participants completed the newly developed NNS-FFQ. A trained research assistant, supervised by a PhD level registered dietitian nutritionist, collected a 24-h dietary recall from the previous day. Between the second and third visit, participants completed one unannounced dietary recall via phone call. At the final visit, participants completed the NNS-FFQ for a second time, and a third 24-h dietary recall was collected.

### 2.2. Development of the Non-Nutritive Sweetener Food Frequency Questionnaire (NNS-FFQ) 

The NNS-FFQ (Online Supplementary Material) is a fully-quantitative tool measuring how often a NNS-containing dietary item is consumed (i.e., never, 1 time per week, 2–3 times per week, etc.) over the past month and how much of the product is consumed each time (i.e., <6 fl oz, 1 tablespoon, 1 cookie, etc.). To create this questionnaire, common sources of NNS were identified [14], and then Nutrition Data System for Research (NDS-R) 2015 was used to characterize each type of food or beverage by the types of NNS used. The first page of the NNS-FFQ is comprised of beverages sweetened with NNS and the second page includes sweetener packets as well as food items, including but not limited to yogurts, ice cream, candy, and chewing gum. Each line of the FFQ represents a unique combination and amount of NNS used. For example, some diet sodas are sweetened with aspartame and acesulfame potassium, while others are sweetened with sucralose and acesulfame potassium. Additionally, the NNS-FFQ identifies products by categories as well as by brand names, allowing participants to more easily identify NNS products they consume. 

The questionnaire gathers data on five NNS: acesulfame potassium, aspartame, saccharin, sucralose, and stevia products that use erythritol as a bulking agent. Erythritol was measured rather than stevia as NDS-R 2015 does not yet report stevia content but does include grams of erythritol in its database. Erythritol is a non-caloric sugar alcohol frequently used as a bulking agent with stevia products. Other sugar alcohols were not included in this analysis, since they do contain nutritive content [19] and are not typically categorized as NNS. While using erythritol for the analysis may present some limitations, it is currently the best option available for the validation of the NNS-FFQ until dietary data on stevia becomes more available. 

Instructions on the questionnaire state that the participant should respond based on intake over the previous month, review each category, and indicate how often each item is consumed and how much is consumed each time. In addition to the categories listed, there is an option for “Other” NNS products for when participants recognize that a product they consume is artificially sweetened but not listed. The NNS-FFQ administration time is between five and 10 minutes, with an additional five minutes of scoring time. Scoring instructions are freely available from the corresponding author upon request.

### 2.3. Dietary Recalls

Dietary recalls were collected by trained graduate-level research assistants. Three recalls were collected on non-consecutive days, including two weekdays and one weekend day, using a multi-pass method. Dietary recalls were analyzed using NDS-R 2015 nutrition analysis software (Nutrition Coordinating Center, University of Minnesota, Minneapolis, MN, USA). For consistency, each participant worked with the same research assistant for all three dietary recalls and the research assistant collecting the recalls was also responsible for the data entry. Dietary recalls were analyzed to determine NNS consumption for the days reported. NDS-R 2015 provides data on saccharin, aspartame, sucralose, acesulfame potassium, and erythritol in products, but currently not for stevia. 

### 2.4. Consumer versus Non-Consumer Analysis

Using a published novel method to categorize NNS consumers and non-consumers [14], participants were identified as NNS consumers if they reported consuming the NNS equivalent of 1 fl oz of diet soda from all foods and beverages. This intake level corresponds to 3 mg acesulfame potassium, 17 mg aspartame, 12 mg saccharin, or 6 mg sucralose. Erythritol was not included in this analysis due to the fact that it is not used in stevia-sweetened soda. 

### 2.5. Statistical Analysis

Descriptive statistics (mean ± standard deviation and frequencies) were used to assess participant demographics and NNS consumption patterns. The data were analyzed for normality using a Shapiro–Wilk test. This analysis determined that total NNS intake was not normally distributed (Online Supplementary Material). Thus, participants with NNS intake greater than ±3 standard deviations from the mean were removed (*n* = 2), giving a final analytical sample of 123. Reproducibility of the NNS-FFQ was assessed by comparing the quantities of each NNS type (and total NNS intake) reported in the NNS-FFQ at time 1 (visit 2) and time 2 (visit 3) using Bland–Altman analyses, Spearman’s correlations (*r_s_*) and paired samples *t*-tests. The comparative validity of the NNS-FFQ was measured by comparing quantities of each NNS type (and total NNS intake) reported in the second administration of the NNS-FFQ to the quantities reported in the participants’ three-day average of their dietary recalls via Bland–Altman analyses, Spearman’s correlations (*r_s_*), and paired samples *t*-tests. When interpreting Bland–Altman plots, an agreement level of 95% was considered acceptable [20,21,22,23]. The second administration of the NNS-FFQ was used in the validity analyses as it measures intake over the past month, and was thus representative of the same time period as the 24-h dietary recalls. Cohen’s κ was used to determine the level of agreement between the two methods for identifying NNS consumers vs. non-consumers. An a priori significance level was set at *p* ≤ 0.05. Statistical analyses were conducted using IBM SPSS statistical analysis software (v. 24 for Mac, 2016, SPSS Inc., Chicago, IL, USA).

## 3. Results

### 3.1. Demographic Characteristics

All enrolled adults completed the study (*n* = 125); however, two outliers were removed giving an analytical sample of 123 adults. Participants were mainly Caucasian (75.6%) with an age range of 18–86 years old. Mean body mass index (BMI) was considered slightly overweight (26.0 kg/m^2^), but 55% of the participants had a normal BMI. Income was widely varied, however the majority of participants had a college degree (79%) (Table 1). Table 2 details NNS consumption patterns, including the number of participants who reported consuming any amount of the five types of NNS based on dietary recalls. 

Because the study population was quite diverse, one-way analysis of variance (ANOVA) tests were run to determine differences in total NNS reported via each assessment method between demographic groups. No statistical differences were found in NNS intake reported in the first or second administration of the NNS-FFQ or dietary recalls based on based on sex, age (ages 18–64 and ages 65+), race (Caucasian and non-Caucasian), and BMI (underweight/normal weight and overweight/obese). Significant differences were detected between groups based on education (high school degree or less and some college or more) for total NNS reported via dietary recalls (*F* = 4.407, *p* < 0.01).

### 3.2. Test–Retest Reproducibility

**Figure 2:**
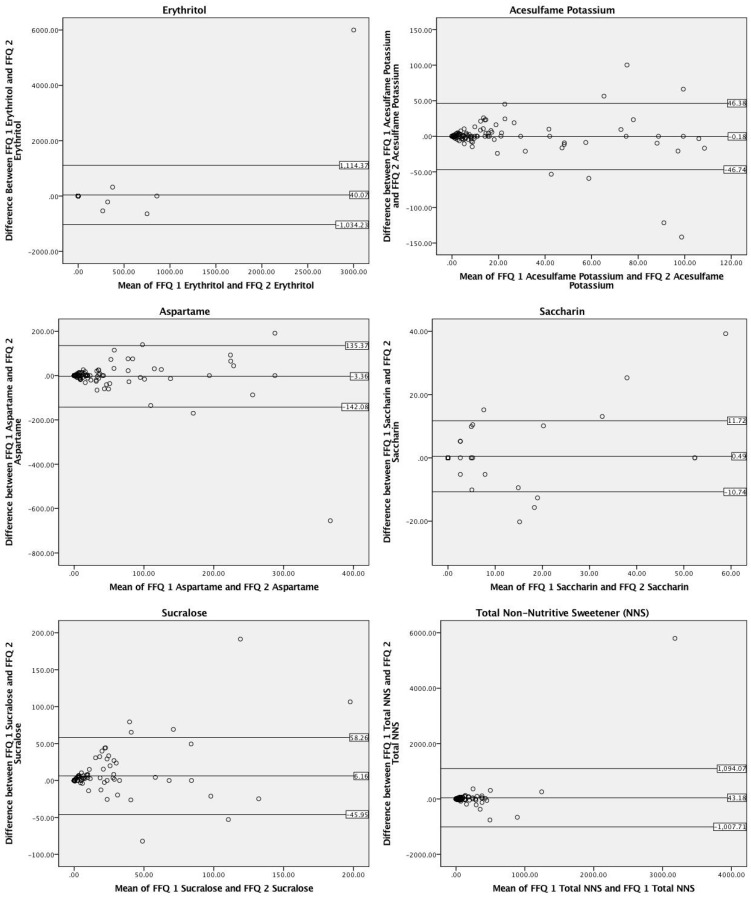
Bland–Altman plots of total and individual non-nutritive sweetener (NNS) mg consumption via two administrations of a NNS food-frequency questionnaire (NNS-FFQ 1 and NNS-FFQ 2). The center line represents the mean difference and the upper and lower lines indicate the mean ± 1.96 times the standard deviation.

When comparing the consumption of individual NNS types between the first and second administration of the NNS-FFQ, Bland–Altman analyses revealed strong agreement for total NNS (99.2%), erythritol (99.2%), and aspartame (96.7%) (Figure 2). Acesulfame potassium (94.3%), saccharin (94.3%), and sucralose (94.3%) agreement levels were slightly below the acceptable 95% value. 

In analyses of correlations and mean differences for reproducibility, all NNS types, as well as total NNS intake, were found to be significantly correlated (Table 3). No significant differences were found between acesulfame potassium, aspartame, saccharin, erythritol, or total NNS intake; however, a small significant mean difference (*p* ≤ 0.05) was found for reported sucralose values. The range of total NNS reported at each administration varied greatly, with the first administration ranging from 0.0 to 6079.1 mg and the second administration ranging from 0.0 to 1221.0 mg. 

Reproducibility analyses were also conducted to detect differences within demographic groups, including sex (male and female), age (18–64 years and ≥65 years), race (white and non-white), education (high school or less and some college or more), and BMI (underweight/normal weight and overweight/obese) (Online Supplementary Material). When comparing reproducibility analyses within demographic groups, there were no significant mean differences among demographic groups and Spearman’s correlations were all significant (range: 0.74–0.93; all *p* ≤ 0.05).

### 3.3. Comparative Validity 

**Figure 3:**
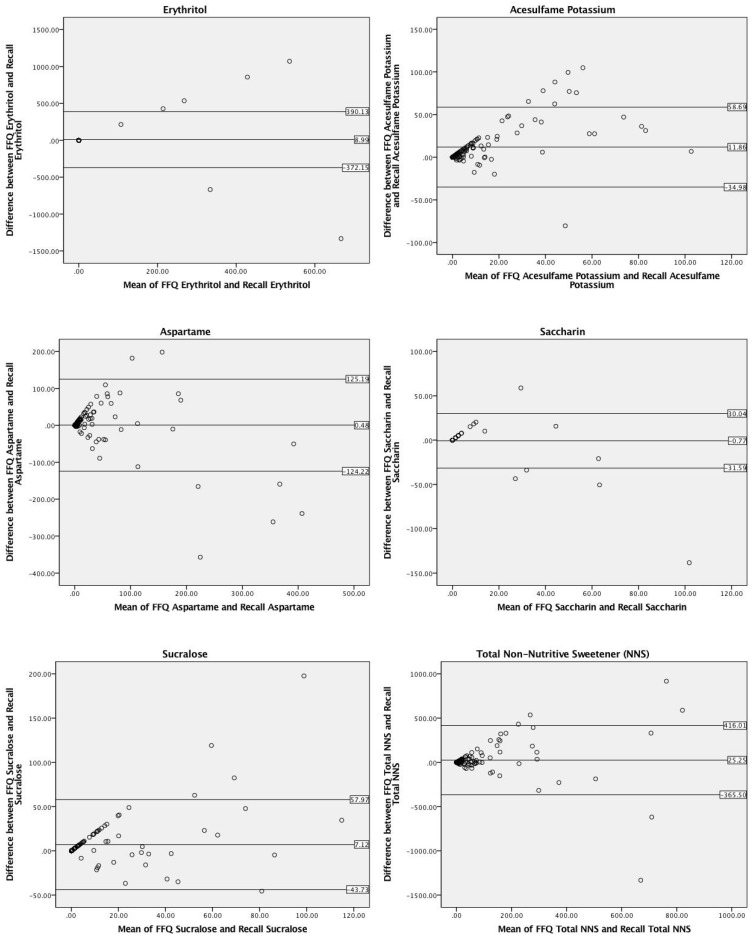
Bland–Altman plots of total and individual non-nutritive sweetener (NNS) mg consumption via a food-frequency questionnaire (FFQ) and dietary recalls (*n* = 123). The center line represents the mean difference and the upper and lower lines indicate the mean ± 1.96 times the standard deviation.

Bland–Altman analyses comparing reported consumption of total and each type of NNS (Figure 3) were conducted to measure agreement of mg intake between the second NNS-FFQ administration and dietary recalls. The second NNS-FFQ was used in this analysis to reflect the same time period during which the dietary recalls were collected. The Bland–Altman analyses revealed agreement levels above the acceptable 95% [20,21,22,23] for total NNS (95.1%), sucralose (95.9%), saccharin (95.9%), erythritol (95.1%) and slightly below for acesulfame potassium (92.7%) and aspartame (94.3%).

When assessing the validity of the NNS-FFQ as compared to the dietary recalls, Spearman’s *r_s_* values for the five sweeteners (acesulfame potassium, aspartame, saccharin, and sucralose) and total NNS ranged from 0.51 to 0.59 (*p* ≤ 0.001) with the exception of erythritol (*r* = −0.03) (Table 4). Significant mean differences were found between the NNS-FFQ and dietary recalls for reported acesulfame potassium values (12.0 ± 27.0 mg, *p* ≤ 0.001). No significant differences were found between aspartame, sucralose, saccharin, erythritol, and total NNS intake. 

Additional comparisons were made to determine if the NNS-FFQ was able to categorize NNS consumers and non-consumers in a similar way to dietary recalls. Based on dietary recall data, 80 participants, or 65.0%, were categorized as NNS consumers compared to 77 participants, or 62.6%, based on the NNS-FFQ data. Cohen’s κ demonstrated a substantial level of agreement between the two methods for identifying NNS consumers vs. non-consumers when looking at reported total NNS consumption, κ = 0.669 (*p* ≤ 0.001) [24]. When examining the κ value for the individual types of NNS, a substantial level of agreement was demonstrated for acesulfame potassium consumers (κ = 0.618, *p* ≤ 0.001), and moderate levels of agreement for aspartame, saccharin, and sucralose (κ = 0.417, 0.601, and 0.517, respectively; all *p* ≤ 0.001). 

Validity analyses were also conducted to detect differences within demographic groups, including sex (male and female), age (18–64 years and ≥65 years), race (white and non-white), education (high school or less and some college or more), and BMI (underweight/normal weight and overweight/obese) (Online Supplementary Material). When comparing validity analyses within demographic groups, there were no significant mean differences based on sex, age, education or BMI, and Spearman’s correlations were all significant (range: 0.44–0.96; all *p* ≤ 0.05). A significant mean difference was detected among white participants (38.4 ± 18.0; *p* = 0.04) and Spearman’s correlation among non-white participants was non-significant (*r* = 0.10).

## 4. Discussion

Based on this investigation, significant correlations were found between total NNS amounts and four individual NNS measured by the NNS-FFQ when compared to amounts measured by participants’ three-day average dietary recalls. This approach of comparing NNS-FFQ amounts to NNS amounts reported in dietary recalls is in agreement with similar dietary assessment validation studies [18]. While there are limitations associated with comparing one self-reported assessment method to another, comparative validity is currently the preferred method for establishing validity before an objective reference measure, such as a dietary biomarker, has been validated. Similar validation studies on FFQ generally consider correlations between 0.4 and 0.7 to be valid [16]. The correlation values for the NNS captured in the NNS-FFQ were between 0.51 and 0.59, with the exception of erythritol. Furthermore, κ values demonstrated moderate (i.e., κ = 0.41–0.60) to substantial (i.e., κ = 0.61–0.80) agreement for identifying consumers and non-consumers for total and all types of NNS [23].

It is important to consider the magnitude of differences found between the measurement methods. Although there were significant differences, the small mass typically consumed may not be clinically relevant. In validity analyses, significant mean differences were found for reported acesulfame potassium values (12.0 mg). This mean difference, while statistically significant, represents about 4 fl oz of diet soda, with 3 mg aspartame in 1 fl oz of diet soda. NNS vary in their sweetness intensity, making the milligram amounts found in each packet different. For instance, a single packet of each sweetener contains 40 mg of saccharin, 50 mg of acesulfame potassium, 11 mg of sucralose, or 9 mg of stevia [3]. Comparing these numbers to the mean difference found in validity analyses shows that all differences were less than half a packet difference, indicating that the statistical differences may not impact clinical outcomes. Similarly, in test–retest reproducibility analyses, significant mean differences were found for reported sucralose values (6.2 mg); however, the mean difference in sucralose is similar to that found in 1 fl oz diet soda, which is about half of what is in one sweetener packet of sucralose. 

While it is noted that reported total NNS intake was not significantly different between the two methods, analyzing individual NNS types is likely to be more valuable in future investigations. One of the major limitations of the current body of literature on NNS is the reliance on analyzing NNS as a broad category rather than as individual compounds [4,25,26,27,28,29]. Given that NNS are understood to be metabolized differently, and thus have different impacts on the human body (e.g., aspartame is metabolized into its constituent parts [30,31], while others are excreted in part or in whole in the urine or feces [31,32]), it is important that research studies measure individual types of NNS. As previously mentioned, the larger mean difference detected between the NNS-FFQ and dietary recalls may be less meaningful due to the wide range of milligram amounts used based on the type of sweetener. 

Erythritol was analyzed to represent stevia products, as it is frequently used as a bulking agent in stevia products. In the validity analysis, erythritol values were not significantly correlated with an *r_s_* value (−0.03) that was lower than the typical range of 0.5 to 0.7 for validity analyses [15]. While this correlation is lower than the other individual sweeteners, this may be attributed to the larger volume at which erythritol is consumed, paired with only a few participants reporting erythritol consumption. Erythritol is generally reported in gram amounts, due to the large mass typically consumed. For instance, 1 stevia sweetener packet contains 3 g, or 3000 mg, of erythritol. Therefore, the mean difference of 9 mg is likely not clinically significant. 

The NNS-FFQ is intended to measure habitual NNS intake over the previous month, whereas dietary recalls collect recent dietary intake. The dietary recalls were collected on non-consecutive days, including two weekdays and one weekend day, which is considered to be more representative of habitual intake than one day alone [33]. However, as dietary recalls measure dietary intake over the previous day and reflect recent intake [15], it would be expected that NNS consumption would not be identical to that reported in the NNS-FFQ, which represents habitual intake. While the NNS-FFQ could be manipulated to measure a shorter or longer time frame, the one-month time frame will likely have higher utility for researchers to measure health outcomes and may also be more manageable for participants to report. It is important to consider the timeframe during which the NNS-FFQ is administered and take into consideration seasonal changes that may take place in individuals’ diets. For example, people may choose to drink more soft drinks during the summer than during the winter. However, the NNS-FFQ is intended to measure intake over the past month, and for this investigation the NNS-FFQ and food recalls were completed within two weeks of each other. FFQ have been studied for their ability to measure shorter [34,35] and longer [24] time frames; therefore, future investigations should consider the ability of the NNS-FFQ to assess a variety of time frames of NNS consumption and to account for seasonal variability. 

The NNS-FFQ has a number of advantages over other methods of dietary assessment, such as 24-h dietary recalls and records. The NNS-FFQ can be used to gauge habitual NNS intake over the previous month, whereas dietary recalls only measure recent intake [16]. The NNS-FFQ can be self-administered, making it convenient and economical to collect large amounts of data in community or epidemiological studies [36] and could be designed to be machine-readable, making it more easily scored for analysis, and thus reducing researcher-associated costs [37]. The scale at which the NNS-FFQ can be used will make it useful for large population-level studies [38].

While there are a number of advantages to using a FFQ, there are also challenges associated with all methods of self-reported data. Due to their subjective nature, self-reported dietary data has known limitations, such as under-reporting of caloric intake [15]. Currently, the “gold standard” for measuring subjective NNS intake is multiple dietary recalls or food records, which are then analyzed with dietary analysis software, such as NDS-R [39]. Dietary assessment for all foods has known challenges, which are compounded in the case of NNS. For example, the FDA does not require specific amounts of NNS used in food products to be reported, making it difficult to quantify NNS intake, even with validated dietary analysis software. Furthermore, if consumers are unable to identify the products they are consuming as artificially sweetened, dietary recalls may fail to capture NNS in consumers’ diets. To help address this limitation, graduate-level research assistants were trained to administer 24-h dietary recalls and enter data using state-of-the-art dietary analysis software. Data from three non-consecutive days including two weekdays and one weekend day was collected. Additionally, to establish rapport and improve data integrity, the same research assistant completed all three dietary recalls and completed the data entry and analysis. Previous investigations have determined the tendency of FFQ to overestimate dietary intake [40]. This analysis also found that the NNS-FFQ was more likely to overestimate NNS intake compared to dietary recalls, with all NNS types being higher based on the NNS-FFQ with the exception of saccharin. This information should be taken into account when using the NNS-FFQ and interpreting results. 

The continually expanding number of products sweetened with NNS is a concern. The FFQ includes an “other” category at the end of the questionnaire for participants to list products not found in the questionnaire. These tended to be generic versions of items already included in the questionnaire and more recently developed products using stevia in this investigation. Twenty participants included information in the “other” category, among which 9 products were matched to related products already included in the FFQ (i.e., generic brands). Eleven participants included items that could not be matched. As previously stated, this analysis used erythritol as a representation for stevia products. When the information was available, NNS quantity was determined using NDS-R or matched the product with similar products using the same combination of sweeteners. In order to accommodate this challenge, this tool will need to be updated periodically to identify the most frequently consumed NNS products. 

While the diversity of this study group is considered a strength as it allows for generalizability across a wide population, it did warrant additional analyses to determine if there were differences in reproducibility and validity results based on demographic characteristics. While there was a significant difference in total NNS consumption between education groups reported in the dietary recalls, no significant difference in validity or reproducibility was found between education groups. Moreover, as there were only seven participants who were 65 years or older, this tool may have limitations in an older adult population; however, the analyses suggest that the NNS-FFQ is valid and reproducible for this age group. Finally, due to a reported difference in total NNS intake between the NNS-FFQ and dietary recalls when analyzing only white participants, future research should thoroughly examine the potential impact of race/ethnicity on the validity of this tool. 

Finally, this tool was developed based on NNS consumption patterns in the US. Therefore, the NNS-FFQ may have limited generalizability outside the US. Future investigations should consider additional versions of the NNS-FFQ that reflect the types of NNS commonly consumed outside the US, as well as commonly consumed brands of NNS food and beverage products. 

NNS consumption has been surrounded by controversy in part due to researchers’ inability to measure NNS intake. Researchers are frequently limited to studying NNS as a whole category or using diet soda as a proxy for NNS consumption, rather than as individual compounds, due to the lack of valid assessment tools able to distinguish between NNS types. Developing methods to measure intake of each NNS type will allow researchers to measure how much of the population consumes NNS and to examine inferentially the potential health outcomes of NNS consumption. 

## 5. Conclusions

This investigation determined that the NNS-FFQ is a reproducible and valid dietary assessment tool that is able to gauge habitual NNS intake patterns relative to 24-h dietary recalls, with the possible exception of erythritol consumption. The NNS-FFQ is a rapid self-report questionnaire that could allow researchers to measure NNS consumption at the individual and population levels among diverse populations. 

## Figures and Tables

**Table 1:** Participant demographic characteristics.

Characteristics	Total Sample (*n* = 123), *n* (%)
Sex	
Male	54 (44)
Female	69 (56)
Mean age ± SD (years)	36.8 ± 16.6
Race/Ethnicity	
Caucasian	93 (75)
Asian/Pacific Islander	17 (14)
African American	7 (6)
Hispanic	3 (2.5)
More than 1 race	3 (2.5)
BMI (kg/m^2^)	
Mean BMI ± SD	26.0 ± 5.7
Underweight (≤18.4)	1 (1)
Normal weight (18.5–24.9)	68 (55)
Overweight (25–29.9)	32 (26)
Obese (≥30)	22 (18)
Education Level	
High-School Graduate	6 (5)
Some College	20 (16)
College Graduate	45 (37)
Graduate School	52 (42)
Household Income ($)	
≤14,999	19 (15)
15,000–29,999	29 (23.5)
30,000–49,999	12 (10)
50,000–99,999	29 (23.5)
≥100,000	22 (18)
No response	12 (10)

**Table 2:** Non-nutritive sweetener (NNS) consumption patterns among 123 adults.

NNS Type	Number of Participants Reporting any Consumption via Dietary Recall *n* (%)	Number of Participants Reporting any Consumption via FFQ 1 *n* (%)	Number of Participants Reporting Any Consumption via FFQ 2 *n* (%)	Cohen’s κ
Acesulfame Potassium	58 (47)	93 (76)	91 (74)	0.681 ***
Aspartame	68 (55)	83 (68)	84 (68)	0.417 ***
Saccharin	7 (6)	21 (17)	17 (14)	0.601 ***
Sucralose	27 (22)	68 (55)	53 (43)	0.517 ***
Erythritol ^a^	2 (2)	5 (4)	5 (4)	n/a ^b^

^a^ Erythritol values were converted from grams to milligrams to compare values across NNS types. ^b^ Cohen’s κ was not included for erythritol due to the inability to classify participants as consumers or non-consumers. *** *p* ≤ 0.001.

**Table 3:** Test–retest reproducibility of a non-nutritive sweetener food frequency questionnaire (NNS-FFQ) (*n* = 123).

NNS Type	NNS-FFQ Time 1 Mean ± SD ^a^ (Median, Range)	NNS-FFQ Time 2 Mean ± SD ^a^ (Median, Range)	Spearman’s Correlation (*r_s_*)	Mean Difference (Mean ± SE) ^b^
Acesulfame Potassium (mg)	18.6 ± 28.9 (5.0, 0.0–132.5)	18.8 ± 32.6 (5.0, 0.0–169.5)	0.81 **	0.2 ± 2.1
Aspartame (mg)	35.3 ± 67.8 (7.0, 0.0–383.1)	38.7 ± 85.1 (7.1, 0.0–694.5)	0.81 **	3.4 ± 6.4
Saccharin (mg)	3.3 ± 11.5 (0.0, 0.0–78.5)	2.9 ± 9.2 (0.0, 0.0–52.3)	0.77 **	0.5 ± 0.5
Sucralose (mg)	18.2 ± 37.6 (2.7, 0.0–250.9)	12.1 ± 28.0 (0.0, 0.0–144.6)	0.81 **	6.2 ± 2.4 *
Erythritol (mg) ^c^	65.3 ± 548.7 (0.0, 0.0–6000.0)	25.3 ± 137.9 (0.0, 0.0–1071.4)	0.78 **	40.1 ± 49.4
Total NNS (mg)	140.8 ± 566.8 (24.0, 0.0–6079.1)	97.6 ± 194.4 (24.6, 0.0–1221.0)	0.92 **	43.8 ± 48.4

^a^ Reported values are means ± standard deviation. ^b^ Mean differences ± standard error according to a paired sample *t* test, slight differences may be noted from the preceding columns due to rounding. ^c^ Erythritol values have been converted from grams to milligrams. * *p* ≤ 0.05, ** *p* ≤ 0.01.

**Table 4:** Comparative validity of a non-nutritive sweetener food frequency questionnaire (NNS-FFQ) as compared to three 24-h dietary recalls (*n* = 123).

Non-Nutritive Sweetener Type	NNS-FFQ 2 Mean ± SD ^a^ (Median, Range)	Dietary Recall Mean ± SD ^a^ (Median, Range)	Spearman’s Correlation (*r_s_*)	Mean Difference (Mean ± SE) ^b^
Acesulfame Potassium (mg)	18.8 ± 32.6 (6.0, 0.0–169.5)	6.8 ± 16.4 (0.0, 0.0–99.3)	0.51 **	12.0 ± 2.4 ***
Aspartame (mg)	38.7 ± 85.1 (7.1, 0.0–694.5)	36.5 ± 97.2 (1.2, 0.0–526.4)	0.59 **	2.2 ± 6.7
Saccharin (mg)	2.9 ± 9.2 (0.0, 0.0–52.3)	3.9 ± 19.6 (0.0, 0.0–170.9)	0.55 **	1.0 ± 1.5
Sucralose (mg)	12.1 ± 28.0 (0.0, 0.0–144.6)	8.0 ± 19.7 (0.0, 0.0–103.7)	0.53 **	4.0 ± 2.1
Erythritol (mg) ^c^	25.3 ± 137.9 (0.0, 0.0–1071.4)	16.3 ± 134.0 (0.0, 0.0–1333.3)	−0.03	9.0 ± 17.5
Total NNS (mg)	97.6 ± 194.4 (25.9, 0.0–1221.0)	72.4 ± 183.6 (8.0, 0.0–1335.7)	0.55 **	25.3 ± 18.0

^a^ Reported values are means ± standard deviation. ^b^ Mean differences ± standard error according to a paired sample *t* test, slight differences may be noted from the preceding columns due to rounding. ^c^ Erythritol values have been converted from grams to milligrams to allow values to be compared across all NNS. ** *p* ≤ 0.01, *** *p* ≤ 0.001.

## Supplementary Materials

The following are available online at http://www.mdpi.com/2072-6643/10/3/334/s1, Figure S1: Artificial Sweetener (Non-nutritive Sweetener) Intake Questionnaire, Figure S2: Correlation scatter plots of total and individual non-nutritive sweetener (NNS) mg consumption via two administrations of a NNS food-frequency questionnaire (NNS-FFQ 1 and NNS-FFQ 2), Figure S3: Correlation scatterplots of total and individual non-nutritive sweetener (NNS) mg consumption via a NNS food-frequency questionnaire (NNS-FFQ 2) and dietary recalls, Table S1: Descriptive statistics of total and individual non-nutritive sweetener (NNS) mg consumption via two administrations of a NNS food-frequency questionnaire (NNS-FFQ 1 and NNS-FFQ 2) and dietary recalls (*n* = 123), Table S2: Reproducibility statistics of total non-nutritive sweetener (NNS) mg consumption from two administrations of a NNS food frequency questionnaire (NNS-FFQ 1 and NNS-FFQ 2) based on demographic characteristics (*n* = 123), Table S3: Validity statistics of total non-nutritive sweetener (NNS) mg consumption from a NNS food frequency questionnaire (NNS-FFQ 2) and three dietary recalls based on demographic characteristics (*n* = 123).
