# Supplementary material for: The Reproducibility and Comparative Validity of a Non-Nutritive Sweetener Food Frequency Questionnaire
Source: Nutrients. 2018 Mar 10;10(3):334. doi: 10.3390/nu10030334 (PMC5872752; doi:10.3390/nu10030334)

Figure S2. Correlation scatterplots of total and individual non-nutritive sweetener (NNS) mg consumption via two administrations of a NNS food-frequency questionnaire (NNS-FFQ 1 and NNS-FFQ 2).

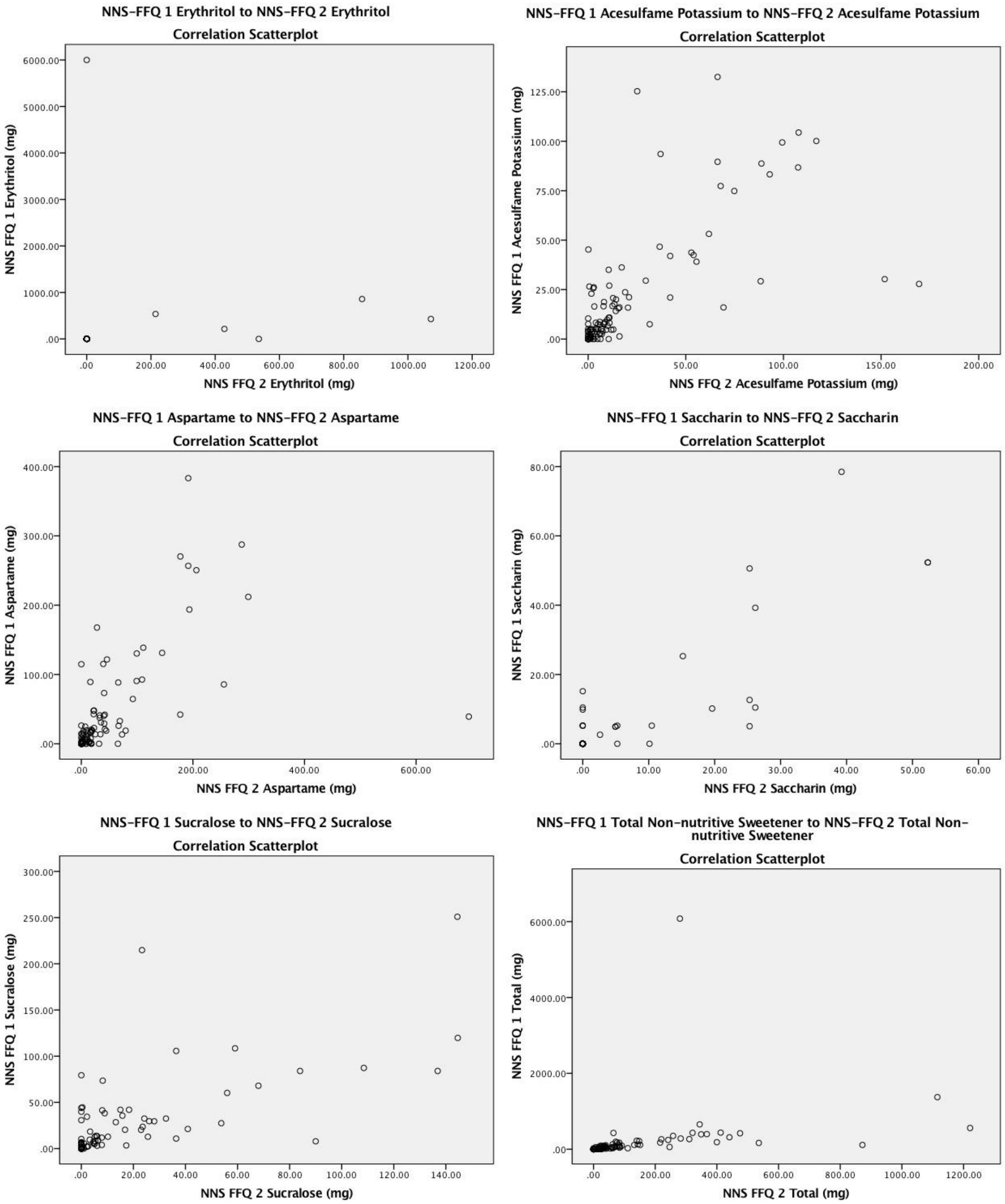

Figure S3. Correlation scatterplots of total and individual non-nutritive sweetener (NNS) mg consumption via a NNS food-frequency questionnaire (NNS-FFQ 2) and dietary recalls.

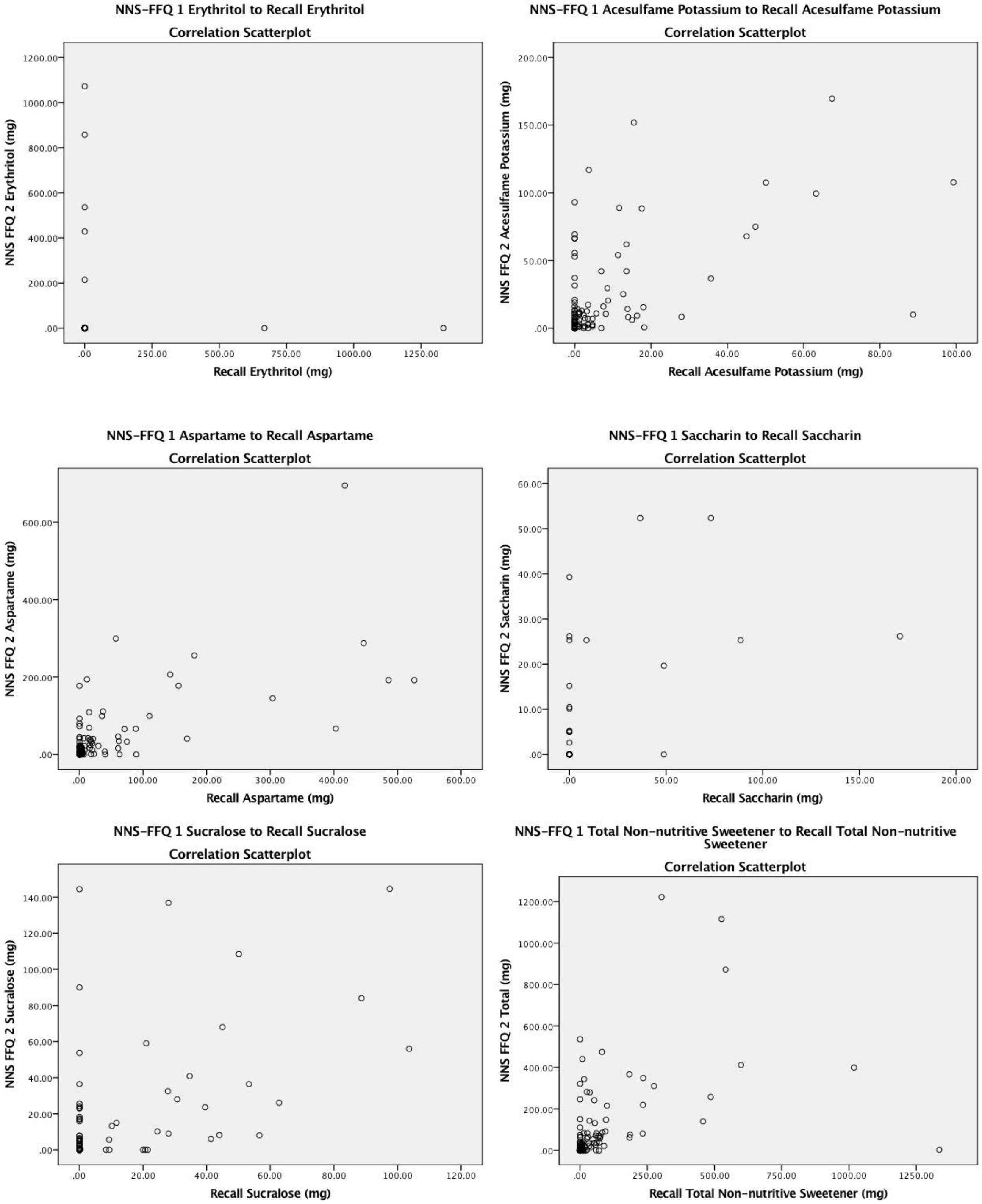

Supplement: Supplementary file 1 [file nutrients-10-00334-s001.zip › Supplemental Material- Correlation Scatterplots.pdf]
