# Supplementary material for: The Reproducibility and Comparative Validity of a Non-Nutritive Sweetener Food Frequency Questionnaire
Source: Nutrients. 2018 Mar 10;10(3):334. doi: 10.3390/nu10030334 (PMC5872752; doi:10.3390/nu10030334)
Supplement: Supplementary file 1 [file nutrients-10-00334-s001.zip › Supplemental Material_Demographic T-Tests.pdf]

Table S2. Reproducibility statistics of total non-nutritive sweetener (NNS) mg consumption from two administrations of a NNS food frequency questionnaire (NNS-FFQ 1 and NNS-FFQ 2) based on demographic characteristics (*n* = 123).

Demographic Characteristics		Total NNS Mean Difference between NNS-FFQ 1 and 2 ± SE	Spearman's Correlations
Sex	Male (*n* = 54)	36.8 ± 24.5	0.93**
	Female (*n* = 69)	16.2 ± 25.8	0.92**
Age	18-64 years (*n* = 116)	46.6 ± 51.3	0.92*
	65+ years (*n* = 7)	12.9 ± 11.1	0.89**
Race	White (*n* = 93)	50.7 ± 63.9	0.93**
	Non-White (*n* = 30)	20.0 ± 11.4	0.74**
Education	High School or Less (*n* = 6)	20.8 ± 45.1	0.77*
	Some College or More (*n* = 117)	46.5 ± 50.8	0.92**
BMI	Underweight/Normal Weight (*n* = 69)	10.4 ± 17.0	0.90**
	Overweight/Obese (*n* = 54)	111.7 ± 107.8	0.93**

*P ≤ 0.05, **P ≤ 0.01

Table S3. Validity statistics of total non-nutritive sweetener (NNS) mg consumption from a NNS food frequency questionnaire (NNS-FFQ 2) and three dietary recalls based on demographic characteristics (*n* = 123).

Demographic Characteristics		Total NNS Mean Difference between NNS-FFQ 2 and Dietary Recalls ± SE	Spearman's Correlations
Sex	Male (*n* = 54)	30.9 ± 19.2	0.69**
	Female (*n* = 69)	101.2 ± 84.5	0.44**
Age	18-64 years (*n* = 116)	23.0 ± 18.8	0.53**
	65+ years (*n* = 7)	62.9 ± 55.2	0.96**
Race	White (*n* = 93)	38.4 ± 18.0*	0.63**
	Non-White (*n* = 30)	15.6 ± 47.9	0.10
Education	High School or Less (*n* = 6)	70.3 ± 112.5	0.75*
	Some College or More (*n* = 117)	30.2 ± 18.0	0.55**
BMI	Underweight/Normal Weight (*n* = 69)	15.9 ± 27.4	0.44**
	Overweight/Obese (*n* = 54)	37.2 ± 21.4	0.64**

*P ≤ 0.05, **P ≤ 0.01
