# Supplementary material for: The Reproducibility and Comparative Validity of a Non-Nutritive Sweetener Food Frequency Questionnaire
Source: Nutrients. 2018 Mar 10;10(3):334. doi: 10.3390/nu10030334 (PMC5872752; doi:10.3390/nu10030334)
Supplement: Supplementary file 1 [file nutrients-10-00334-s001.zip › OSM - NNS FFQ.pdf]

Artificial Sweetener (Non-nutritive Sweetener) Intake Questionnaire

Instructions:

In the past month, please indicate your response for each food or beverage item by marking an "X" in the box for "how often" and "how much each time".

1. Indicate how often you consumed the following items. For example, if you drank diet soda 5 times per week, mark 4-6 times per week.
2. Indicate the approximate amount of each item you consumed each time.
   For example, if you drank 1 cup of diet soda each time, mark 1 cup under "how much each time".
3. Count packets of artificial sweeteners added to foods/beverages at the top of page 2 in the artificial sweetener packet category.
4. Please complete **both** the front and back of the questionnaire.

Participant ID: _______________

Date: _______________

Beverages		HOW OFTEN (MARK ONE)							HOW MUCH EACH TIME (MARK ONE)				
		Never or <1x/week	1x/ week	2-3x/ week	4-6x/ week	1x/day	2x/day	3x/day	<6 fl oz/ ¾ cup	8 fl oz/ 1 cup	12 fl oz/ 1½ cups	16 fl oz/ 2 cups	>20 fl oz/ 2½ cups
Flavored Water	Gatorade G2, Propel Zero	○	○	○	○	○	○	○	○	○	○	○	○
	**Sugar-Free** sparkling or carbonated water	○	○	○	○	○	○	○	○	○	○	○	○
	**Diet** Tonic water	○	○	○	○	○	○	○	○	○	○	○	○
	VitaZest, Fruit2O, **Sugar-Free** flavored water	○	○	○	○	○	○	○	○	○	○	○	○
Juice or Flavored Drink	**Light** Tang, Crystal light (packets for water bottle), **Sugar-Free** Kool-Aid (dry mix), Country Time Light Lemonade (dry mix),	○	○	○	○	○	○	○	○	○	○	○	○
	Ocean Spray **Lightstyle** Juice, Orchard **Light** Cranberry Juice, Tropicana Twister **Light**, **Diet** Snapple	○	○	○	○	○	○	○	○	○	○	○	○
	Ocean Spray **Light** or **light** cranberry juice	○	○	○	○	○	○	○	○	○	○	○	○
Diet Soda Pop/ Soft Drink	Shasta, Rite Pure Zero, Coke *with* Splenda, **Diet** 7UP	○	○	○	○	○	○	○	○	○	○	○	○
	**Diet** Pepsi: Wild Cherry, Vanilla, Lime, or Max<br>**Diet** Coke: Vanilla, Lime, or Cherry, Coke Zero	○	○	○	○	○	○	○	○	○	○	○	○
	Fresca, FantaZero, Tropicana Twister **Light**, **Diet** Sunkist Orange, Sprite Zero, Pibb Zero, **Diet** Mountain Dew Code Red, **Diet** Mello Yello, **Diet** Cherry 7UP, **Diet** Barq's Root Beer, **Diet** A&W Root Beer	○	○	○	○	○	○	○	○	○	○	○	○
	**Diet** Mountain Dew, Pepsi Next	○	○	○	○	○	○	○	○	○	○	○	○
	Zevia soda	○	○	○	○	○	○	○	○	○	○	○	○
	**Diet** Sunkist fruit flavors (other than orange), **Diet** Sundrop, **Die**t Schweppes Ginger Ale, **Diet** Pepsi Twist/regular/caffeine free, **Diet** Mug, **Diet** Mr. Pibb, **Diet** Dr. Pepper, **Diet** Crush, **Diet** Canada Dry Ginger Ale, **Diet** A&W Cream Soda	○	○	○	○	○	○	○	○	○	○	○	○
Diet Tea	**Diet** Bottled Lipton Tea, Crystal Light Instant	○	○	○	○	○	○	○	○	○	○	○	○
	Crystal Light decaffeinated, **Diet** Nestea	○	○	○	○	○	○	○	○	○	○	○	○
	**Diet** Lipton Instant Iced Tea Mix	○	○	○	○	○	○	○	○	○	○	○	○
Chocolate Drink	**Sugar-Free** Nestle Nesquik or **Sugar-Free** Swiss Miss	○	○	○	○	○	○	○	○	○	○	○	○
Coffee	Frappuccino **Light**, ready to drink	○	○	○	○	○	○	○	○	○	○	○	○
Energy Drink	**Sugar-Free Versions:** Sobe, No Fear, AMP, Rockstar, Monster, Venom Majave	○	○	○	○	○	○	○	○	○	○	○	○
	**Sugar-Free** Red Bull	○	○	○	○	○	○	○	○	○	○	○	○
Protein Drink	Carnation Instant Breakfast **No Sugar Added**, Slim-fast Easy Digest, Slim-fast Optima	○	○	○	○	○	○	○	○	○	○	○	○
	Slim-fast Meal **Low Carb**	○	○	○	○	○	○	○	○	○	○	○	○

Virginia Polytechnic Institute and State Univeristy, 2017

Please continue to the next page to complete this questionnaire.

Please circle each item you consumed.

Artificial Sweetener (Non-nutritive Sweetener) Intake Questionnaire

	HOW OFTEN (MARK ONE)							HOW MUCH EACH TIME (MARK ONE)				
**Artificial Sweetener Packets**	**Never or <1x/week**	**1x/ week**	**2-3x/ week**	**4-6x/ week**	**1x/day**	**2x/day**	**3x/day**	**<½ packet**	**1 packet**	**1½ packets**	**2 packets**	**3+ packets**
Sweet 'N Low Powder or Sugar Twin (Saccharin) - Pink Packets	○	○	○	○	○	○	○	○	○	○	○	○
Equal or NutraSweet The Original (Aspartame) - Blue Packets	○	○	○	○	○	○	○	○	○	○	○	○
Splenda (Sucralose) - Yellow Packets	○	○	○	○	○	○	○	○	○	○	○	○
Sweet One (Acesulfame Potassium)	○	○	○	○	○	○	○	○	○	○	○	○
Truvia (Stevia)	○	○	○	○	○	○	○	○	○	○	○	○
Stevia In The Raw Packets (Stevia)	○	○	○	○	○	○	○	○	○	○	○	○
**Yogurt and Ice Cream**	**Never or <1x/week**	**1x/ week**	**2-3x/ week**	**4-6x/ week**	**1x/day**	**2x/day**	**3x/day**	**<4 oz/ ½ cup**	**8 oz/ 1 cup**	**12 oz/ 1½ cups**	**16 oz/ 2 cups**	**>20 oz/ 2½ cups**
Breyers **Light** Yogurt	○	○	○	○	○	○	○	○	○	○	○	○
Yoplait **Light** Thick & Creamy	○	○	○	○	○	○	○	○	○	○	○	○
Blue Bunny **Light** Yogurt	○	○	○	○	○	○	○	○	○	○	○	○
Dannon **Light** & Fit or Activia **Light** Yogurt	○	○	○	○	○	○	○	○	○	○	○	○
Yoplait **Light** Fat Free Yogurt	○	○	○	○	○	○	○	○	○	○	○	○
**Light Ice Creams:** Edy's, Dreyer's, Blue Bell, Baskin-Robbins, Popsicle	○	○	○	○	○	○	○	○	○	○	○	○
Blue Bunny **Light** Ice Cream	○	○	○	○	○	○	○	○	○	○	○	○
**Sugar-Free** Creamsicle or Dreamsicle	○	○	○	○	○	○	○	○	○	○	○	○
**Cookies and Bars**	**Never or <1x/week**	**1x/ week**	**2-3x/ week**	**4-6x/ week**	**1x/day**	**2x/day**	**3x/day**	**<1 cookie or bar**	**1 cookie or bar**	**2 cookies or bars**	**3 cookies or bars**	**4+ cookies or bars**
**Sugar-Free Versions:** Pillsbury, Nabisco, Murray Cookies, Slim-fast Snack/Meal Bar	○	○	○	○	○	○	○	○	○	○	○	○
**Sugar-Free Versions:** Quaker Chewy, Power Bar, Snackwell, Tastykake Sensables, Pepperidge Farm Milano, Slim-fast Meal Bar (Chocolate Peanut Caramel)	○	○	○	○	○	○	○	○	○	○	○	○
**Candy**	**Never or <1x/week**	**1x/ week**	**2-3x/ week**	**4-6x/ week**	**1x/day**	**2x/day**	**3x/day**	**<1 piece**	**1 piece**	**2 pieces**	**3 pieces**	**4+ pieces**
**Sugar-Free** Chewing gum	○	○	○	○	○	○	○	○	○	○	○	○
**Sugar-Free Versions:** Twizzler, Jolly Rancher, Fifty 50, Sweet 'N Low Fruit Splash, Werther's	○	○	○	○	○	○	○	○	○	○	○	○
**Sugar-Free** Chocolate (Crystal Light Candy, DeMet's, Dove, Fifty 50, York Peppermint Patty), **Sugar-Free** Gum drops, Gummy worms/bears	○	○	○	○	○	○	○	○	○	○	○	○
**Jello and Pudding**	**Never or <1x/week**	**1x/ week**	**2-3x/ week**	**4-6x/ week**	**1x/day**	**2x/day**	**3x/day**	**½ cup**	**1 cup**	**1½ cups**	**2 cups**	**2½+ cups**
**Sugar-Free** or **Reduced-Calorie** Jell-O Pudding (cooked or instant)	○	○	○	○	○	○	○	○	○	○	○	○
**Sugar-Free** or **Reduced-Calorie** Jell-O Pudding Cup, Hunt's Snack Pack Pudding Cup (ready to eat)	○	○	○	○	○	○	○	○	○	○	○	○
**Sugar-Free** or **Reduced-Calorie** Jell-O Gelatin (prepared from dry mix)	○	○	○	○	○	○	○	○	○	○	○	○
**Sugar-Free** or **Reduced-Calorie** Jell-O Gelatin Cup (ready to eat)	○	○	○	○	○	○	○	○	○	○	○	○
**Sugar-Free** or **Reduced-Calorie** Hunt's Snack Pack Gelatin Cup (ready to eat)	○	○	○	○	○	○	○	○	○	○	○	○
**Jelly**	**Never or <1x/week**	**1x/ week**	**2-3x/ week**	**4-6x/ week**	**1x/day**	**2x/day**	**3x/day**	**1 tsp**	**2 tsps**	**1 tbsp**	**2 tbsps**	**3+ tbsps**
**Sugar-Free** Jelly with sucralose (Smucker's Sugar-Free with Splenda, Great Value Sugar-Free Preserves)	○	○	○	○	○	○	○	○	○	○	○	○
**Sugar-Free** Jelly with stevia or Truvia (Smucker's Sugar-Free with Truvia)	○	○	○	○	○	○	○	○	○	○	○	○
**Coffee Creamer**	**Never or <1x/week**	**1x/ week**	**2-3x/ week**	**4-6x/ week**	**1x/day**	**2x/day**	**3x/day**	**1 tsp**	**2 tsps**	**1 tbsp**	**2 tbsps**	**3+ tbsps**
**Sugar-Free** Flavored Creamer (powder or liquid), **Sugar-Free** Coffeemate or International Delight	○	○	○	○	○	○	○	○	○	○	○	○
**Other Products with Artificial Sweeteners:**	**Never or <1x/week**	**1x/ week**	**2-3x/ week**	**4-6x/ week**	**1x/day**	**2x/day**	**3x/day**	**½ serving**	**1 serving**	**1½ serving**	**2 serving**	**3 servings**
**Other (List):**	○	○	○	○	○	○	○	○	○	○	○	○

Virginia Polytechnic Institute and State Univeristy, 2017
