# Supplementary material for: The Reproducibility and Comparative Validity of a Non-Nutritive Sweetener Food Frequency Questionnaire
Source: Nutrients. 2018 Mar 10;10(3):334. doi: 10.3390/nu10030334 (PMC5872752; doi:10.3390/nu10030334)
Supplement: Supplementary file 1 [file nutrients-10-00334-s001.zip › Supplemental Material - Descriptive Statistics.pdf]

Table S1. Descriptive statistics of total and individual non-nutritive sweetener (NNS) mg consumption via two administrations of a NNS food-frequency questionnaire (NNS-FFQ 1 and NNS-FFQ 2) and dietary recalls (n = 123).

Non-nutritive Sweetener Type	Mean (mg)	Std. Deviation	Median	Minimum	Maximum	Skewness		Kurtosis		Shapiro-Wilk Tests of Normality	
						Statistic	Std. Error	Statistic	Std. Error	Statistic	Sig
NNS-FFQ 1 Erythritol (mg)	65.3	548.7	0.00	0.0	6000.0	10.6	0.2	114.8	0.4	0.1	≤0.001
NNS-FFQ 1 Acesulfame Potassium (mg)	18.6	28.9	5.0	0.0	132.5	2.2	0.2	4.2	0.4	0.7	≤0.001
NNS-FFQ 1 Aspartame (mg)	35.3	67.8	7.0	0.0	383.3	2.9	0.2	8.7	0.4	0.6	≤0.001
NNS-FFQ 1 Saccharin (mg)	3.3	11.5	0.0	0.0	78.5	4.6	0.2	22.0	0.4	0.3	≤0.001
NNS-FFQ 1 Sucralose (mg)	18.2	37.6	2.7	0.0	250.9	3.7	0.2	17.4	0.4	0.5	≤0.001
NNS-FFQ 1 Total NNS (mg)	140.8	566.8	24.0	0.0	6079.1	9.7	0.2	100.9	0.4	0.2	≤0.001
NNS-FFQ 2 Erythritol (mg)	25.3	137.9	0.0	0.0	1071.4	6.1	0.2	39.1	0.4	0.2	≤0.001
NNS-FFQ 2 Acesulfame Potassium (mg)	18.8	32.6	5.0	0.0	169.5	2.5	0.2	6.1	0.4	0.6	≤0.001
NNS-FFQ 2 Aspartame (mg)	38.7	85.1	7.1	0.0	694.5	4.7	0.2	29.7	0.4	0.5	≤0.001
NNS-FFQ 2 Saccharin (mg)	2.9	9.2	0.0	0.0	52.3	3.8	0.2	15.5	0.4	0.4	≤0.001
NNS-FFQ 2 Sucralose (mg)	12.1	28.0	0.0	0.0	144.6	3.3	0.2	11.4	0.4	0.5	≤0.001
NNS-FFQ 2 Total NNS (mg)	97.6	194.4	24.6	0.0	1221.0	3.7	0.2	15.8	0.4	0.5	≤0.001
Recall Erythritol (mg)	16.3	134.0	0.0	0.0	1333.3	8.9	0.2	82.0	0.4	0.1	≤0.001
Recall Acesulfame Potassium (mg)	6.8	16.4	0.0	0.0	99.3	3.7	0.2	14.8	0.4	0.4	≤0.001
Recall Aspartame (mg)	36.5	97.2	1.2	0.0	526.4	3.7	0.2	13.6	0.4	0.2	≤0.001
Recall Saccharin (mg)	3.9	19.6	0.0	0.0	170.9	6.5	0.2	47.5	0.4	0.5	≤0.001
Recall Sucralose (mg)	8.0	19.7	0.0	0.0	103.7	3.0	0.2	9.6	0.4	0.5	≤0.001
Recall Total NNS (mg)	72.3	183.6	7.7	0.0	1335.7	4.5	0.2	24.1	0.4	0.4	≤0.001
